# Bisphenol A Induces Histopathological, Hematobiochemical Alterations, Oxidative Stress, and Genotoxicity in Common Carp (*Cyprinus carpio* L.)

**DOI:** 10.1155/2022/5450421

**Published:** 2022-01-28

**Authors:** Gulnaz Afzal, Hafiz Ishfaq Ahmad, Riaz Hussain, Adil Jamal, Shumaila Kiran, Tarique Hussain, Saba Saeed, Mehr un Nisa

**Affiliations:** ^1^Department of Zoology, The Islamia University of Bahawalpur, Bahawalpur, Pakistan; ^2^Department of Animal Breeding and Genetics, University of Veterinary and Animal Sciences, Lahore, Pakistan; ^3^Department of Pathology, Faculty of Veterinary and Animal Sciences, The Islamia University of Bahawalpur, Bahawalpur, Pakistan; ^4^Sciences and Research, College of Nursing, Umm Al Qura University, Makkah 715, Saudi Arabia; ^5^Department of Applied Chemistry, Government College University, Faisalabad, Pakistan; ^6^Animal Sciences Division, Nuclear Institute for Agriculture and Biology College, Pakistan Institute of Engineering and Applied Sciences (NIAB-C, PIEAS), Jhang Road, Faisalabad 38000, Pakistan; ^7^Institute of Physics, The Islamia University of Bahawalpur, Bahawalpur, Pakistan

## Abstract

Bisphenol A (BPA) is one of the environmental endocrine disrupting toxicants and is widely used in the industry involving plastics, polycarbonate, and epoxy resins. This study was designed to investigate the toxicological effects of BPA on hematology, serum biochemistry, and histopathology of different organs of common carp (*Cyprinus carpio*). A total of 60 fish were procured and haphazardly divided into four groups. Each experimental group contained 15 fish. The fish retained in group A was kept as the untreated control group. Three levels of BPA 3.0, 4.5, and 6 mg/L were given to groups B, C, and D for 30 days. Result indicated significant reduction in hemoglobin (Hb), lymphocytes, packed cell volume (PCV), red blood cells (RBC), and monocytes in a dose-dependent manner as compared to the control group. However, significantly higher values of leucocytes and neutrophils were observed in the treated groups (*P* < 0.05). Results on serum biochemistry revealed that the quantity of glucose, cholesterol, triglycerides, urea, and creatinine levels was significantly high (*P* < 0.05). Our study results showed significantly (*P* < 0.05) increase level of oxidative stress parameters like reactive oxygen species (ROS) and thiobarbituric acid reactive substances (TBARS) and lower values of antioxidant enzymes (superoxide dismutase (SOD), catalase (CAT), peroxidase (POD) in treated groups (4.5 mg/L and 6 mg/L)) in the brain, liver, gills, and kidneys. Our study depicted significant changes in erythrocytes (pear shaped erythrocytes, leptocytes, microcytes, spherocytes, erythrocytes with broken, lobed, micronucleus, blabbed, vacuolated nucleus, and nuclear remnants) among treated groups (4.5 mg/L and 6 mg/L). Comet assay showed increased genotoxicity in different tissues including the brain, liver, gills, and kidneys in the treated fish group. Based on the results of our experiment, it can be concluded that the BPA exposure to aquatic environment is responsible for deterioration of fish health, performance leading to dysfunction of multiple vital organs.

## 1. Introduction

BPA is an estrogenic endocrine distorting chemical being used in manufacturing of polycarbonate and epoxy resins [1–3]. It is also present in dental sealants, water and baby bottles, food and beverage packaging, paper coatings, flame retardants, and adhesives [4, 5]. In 2011, by considering the BPA lethality, The European Commission (EC) has banned its use in the production of polycarbonate and infant feeding bottles. Despite of regulations on BPA, a variety of BPA analogues are being extensively manufactured and applied worldwide. However, predominantly BPA is still one of the most analogues that contaminate aquatic ecosystems causing health-related threats to the aquatic life [6–8]. BPA discharges into water environment not only from daily useable but also from landfill sites and waste water treatment plants [9]. Considering the effects of anthropogenic and local attributes of aquatic ecosystems, BPA toxicity has been reached at maximum level with geometric means. According to meta-analysis, the recorded values of BPA in fresh water is 42.3 (63,640) ng L^−1^, in brackish water is 28.6 (5,100) ng L^−1^, and in sea water is 17.7 (1,918) ng L^−1^ [10]. Consequently, it is difficult for aquatic organism such as tiny fish, plants, spineless creatures, and vertebrates to escape from the harmful impact of BPA [11, 12]. In human, BPA exposure below the level of average exposure (50 to 4 *μ*g/Kg weight/day) is more lethal as compared to high unpredicted doses [13, 14]. BPA, like other intestinal phenols, and glucuronic acid have ability to get absorbed and ingested by human gastrointestinal tract and liver cells and may be excreted in urine [15, 16]. Furthermore, due to estrogenic activity, its pre- and postnatal exposure can decrease serum testosterone and erythropoietin production level in animals which results in increase in destruction of red blood cells by decreasing the concentration of hemoglobin [17–19]. It can also be stored in adipose tissues due to its lipophilic nature [20–22]. Among aquatic animals, fish are highly sensitive to different pollutants including BPA which is mainly absorbed through the skin, gills, and alimentary route and get absorbed into the body tissues by disrupting the physiological and biochemical processes. A number of studies regarding effects of BPA on the growth, behavior, morphological characters, genotoxicity, biochemical, and histological changes in fish were reported [23–27]. BPA may also change the gene expression pattern throughout the development of body organs and is strongly influenced by both genetic and environmental factors [28, 29]. It can cause the alteration of calcium homeostasis by inhibiting the calcium regulating hormones in goldfish [30]. It may also increase the lymphocyte production at high concentrations of 500–1,000 mg/L which may ultimately inhibit macrophage production in goldfish [31]. Liver and kidneys of fish can also be damaged with the increase of creatinine level when they were subjected to the continuous exposure of BPA [32, 33]. Moreover, BPA contaminated fish have also been served as the bioindicators of aquatic environments [34]. In environmental studies, the detection of different biomarkers in response of adverse effects of BPA has been proven as a sensitive and reliable end point [35].

Reports documented sublethal toxicity effects of BPA in fresh water carp (*Aristichthys nobis*) including hematological, biochemical, erythrocytes, organs, and nuclear changes [12]. Addressed information of bisphenol A persistent toxicity is not sufficient regarding antioxidant enzymes, oxidative stress markers, erythrocytes, and nuclear changes in *Cyprinus carpio*. Studies have highlighted that investigation of hematobiochemical parameters and morphological changes in erythrocyte of fish are reliable and useful tools for monitoring to toxic effects of different environmental pollutants [36–38]. Therefore, current study is aimed at exploring the potential toxic effects of BPA on the blood, brain, liver, gills, and kidneys of *C. carpio*.

In light of the abovementioned observations, the current study was planned to investigate the toxic effects of BPA on the common carp (*Cyprinus carpio*). *C. carpio* is an important fish used as food, having high fecundity/hatchability rate and easy to culture in intensive and semi-intensive cultures [39]. More importantly, *C. carpio* farming employs more than 400.000 people in Pakistan [40]. According to literature, BPA effects specifically on *C. carpio* are scarce. Therefore, we select the fresh water fish common carp as a model animal to determine the toxicological effects of BPA at low levels. Current study is aimed at exploring the alterations in histopathological, hematobiochemical, oxidative stresses, and antioxidants pattern in common carp subjected to bisphenol A exposure. We also highlighted the erythrocytes changes and genotoxicity (nuclear changes) in common carp exposure to BPA toxin.

## 2. Experimental

### 2.1. Ethical Statement

The present study was performed in the laboratories of Department of Zoology and Department of Pathology (Faculty of Veterinary and Animal Sciences), The Islamia University of Bahawalpur. All the standard protocols for the preparation of chemicals and reagents were used in the whole experiment. The ethics of animals handling provided by the Institutional Bioethics Committee (IBC) of The Islamia University of Bahawalpur, Pakistan, were strictly followed.

### 2.2. Chemicals

All the analytical grade chemicals used in this research were attained from Merck (Germany) and Sigma-Aldrich, (St. Louis, Missouri, USA). The crystalline white powder of BPA (CAS registry No. 80-05-7, purity of 99%) was acquired from commercial scientific store at Lahore, Pakistan.

### 2.3. Experimental Design

A total of 60 common carps (*Cyprinus carpio*), active and apparently healthy, with the weight ranging from 200 to 250 g were obtained from the local fisheries complex of district Bahawalpur, Pakistan. All fish were brought to laboratory in plastic bags with appropriate hatchery water and oxygen and then left to glass aquaria (25^″^*L* × 45^″^*W* × 35^″^*H*) for acclimatization under standard laboratory conditions at ±25°C temperature for 15 days. The pH of water, different other water quality profile, was determined prior to start of experimental research, and aerators were maintained in the all aquaria. Following acclimatization, all fish were randomly divided into four groups and were kept in 100 L water in glass aquaria (15 fish in each) with constant photoperiod of 12/12 h day-night cycle, where group A was the control group while B-D were the treated groups. All the aquaria were supplied with oxygenators to maintain sufficient supply of oxygen to experimental fish. Different sublethal concentrations of BPA (3, 4.5 and 6 mg/L) were selected and poured in each aquarium in accordance with the previous study [41]. After every third day, water of each aquarium was replaced with the fresh water. All the fish were daily given 30 CP (crude protein), and the daily feed intake was set to 3% of the fish body weight. All behavioral changes, clinical signs, and mortality rate were observed throughout the experimental period.

### 2.4. Hematobiochemical Parameters

At day 30, blood samples from caudal vein of each treated and control fish were collected by using 26-gauge hypodermic needle and then kept in anticoagulant EDTA coated glass tubes for processing the hematology [42]. All the experimental fish were anaesthetized using clove oil (4.5 mg/L) to reduce the stress and for collection of blood samples. Various blood parameters including total erythrocyte count, toal and differential leukocyte count, and pack cell volume were measured [43]. For serum separation, blood samples placed in anticoagulant EDTA coated tubes were first placed in ice and then centrifuged at 2500 × g for 10 mins. The supernatant were aliquoted for analyzing the serum biochemical parameters including urea, albumin, creatinine, glucose, cholesterol, and triglycerides by following the protocols of available commercial kits (M/S Randox Company) with the help of chemistry analyzer [44, 45].

### 2.5. Histopathological Analysis

For histopathological analysis, all fish were given anesthesia with isoflurane in a separate chamber for dissection. The vital organs such as brain, gills, heart, kidneys, and liver were immediately removed from all fish groups and handled with the standard protocol devised by [46]. Concisely, samples were rinsed in isotonic saline solution and preserved in the neutral buffered formaldehyde (pH 7.2) solution. After preservation of all tissue samples, ascending ordered alcoholic solutions were used to dehydrate them and finally embed them in the paraffin wax. A rotatory microtome (Shandon Finesse, Italy) was used to slice the 4-5 *μ*m thick sections from all the tissues. All sections were kept to dry on a slide warmer at 37°C, deparaffinized in xylene, and again set to dehydrate it through a series of ascending ordered alcohol solutions. Finally, all sections were stained with standard hematoxylin and eosin (H&E) methods, cleared in xylene again, and mounted in DPX mountant medium. Sections of all the test specimens were observed under light microscope (Leica, Germany).

### 2.6. Genotoxicity (Nuclear Damage) Evaluation

For morphological and nuclear changes in erythrocytes of treated groups, fine thin blood film was prepared without anticoagulants. To study the erythrocytes morphology, approximately 1500 red blood cells were observed from each fish using light microscope [47], We used comet assay to study the nuclear damage in tissues including brain, liver, gills, and kidneys [48, 49]. Electrophoresis was performed at 25 V for 30 min [50]. Following electrophoresis, slides were neutralized with chilled 0.5 M Tris buffer (ph 7.5). Finally, the ethidium bromide-stained slides were visualized under fluorescence microscope at ×400 magnification power.

### 2.7. Oxidative Stress Parameters and Antioxidant Enzyme Estimation

To study the biochemical parameters, fish of each control and treatment groups were dissected at day 30. Brain, liver, gills, and kidneys of dissected fish were placed in ice chilled saline solution. Each tissue was prepared for estimation of reactive oxygen species (ROS), reduced glutathione (GSH), thiobarbituric acid reactive substances (TBARS), and antioxidant enzymes including peroxidase (POD), catalase (CAT), and superoxide dismutase (SOD). Oxidative stress parameters in the brain, liver, kidney, and gills were estimated following the reported studies earlier including ROS [51], GSH [52], and TBARS [53]. Antioxidant enzymes in the brain, liver, gills, and kidneys of treated and control fish were determined according to documented protocol earlier including POD, CAT [54], and SOD [55].

### 2.8. Statistical Analysis

Data was analyzed by ANOVA using SPSS (SPSS Inc., Illinois, USA) program. Mean ± SE values for hematological, biochemical, nuclear changes in erythrocytes, oxidative stresses, and antioxidant enzymes among control and treated groups were compared by Tukey's test. Calculated values of *P* < 0.05 were considered statistically significant.

## 3. Results

### 3.1. Physical and Blood Biochemical Responses

No clinical signs/abnormalities and mortality were observed in the untreated control group-A. The treated groups with low to high doses of BPA (3, 4.5, 6 mg/L) showed different mild to severe (dose and time dependent) physical responses like loss of equilibrium, operculum movement, faintness, changed skin color (black spots on body surface), a regular secretion of mucosa from gills and mouth, tremor of fins, jerking with uneven swimming, lying on one side while swimming, air gulping, and bulging of eyes. Severity of different clinical and behavioral signs including loss of equilibrium, tremor of fins, operculum movement, mucous secretion, and dark skin color was increased with increased time for the fish exposed to 4.5 mg/L and 6 mg/L of BPA.

In hematological analysis of the controlled group, the values of all parameters including hemoglobin, erythrocytes, leucocytes, lymphocytes, packed cell volume (PCV), neutrophils, and monocytes were found normal. However, in BPA exposed groups, the values of hemoglobin, erythrocytes, lymphocytes, PCV, and monocytes were found significantly decreased depending on the dose concentration when compared with the control group (*P* < 0.05), while gradually increased values of leucocytes and neutrophils were observed in treated groups when compared to the control group (Table 1). According to biochemical analysis of BPA exposed groups, the values of glucose, cholesterol, triglycerides, creatinine, and urea were observed significantly high as compared to the control group (*P* < 0.05) while albumin protein was observed gradually decreased in dose-dependent manner as compared to the control group (Table 1).

### 3.2. Gross and Histopathological Studies

**Figure 1:**
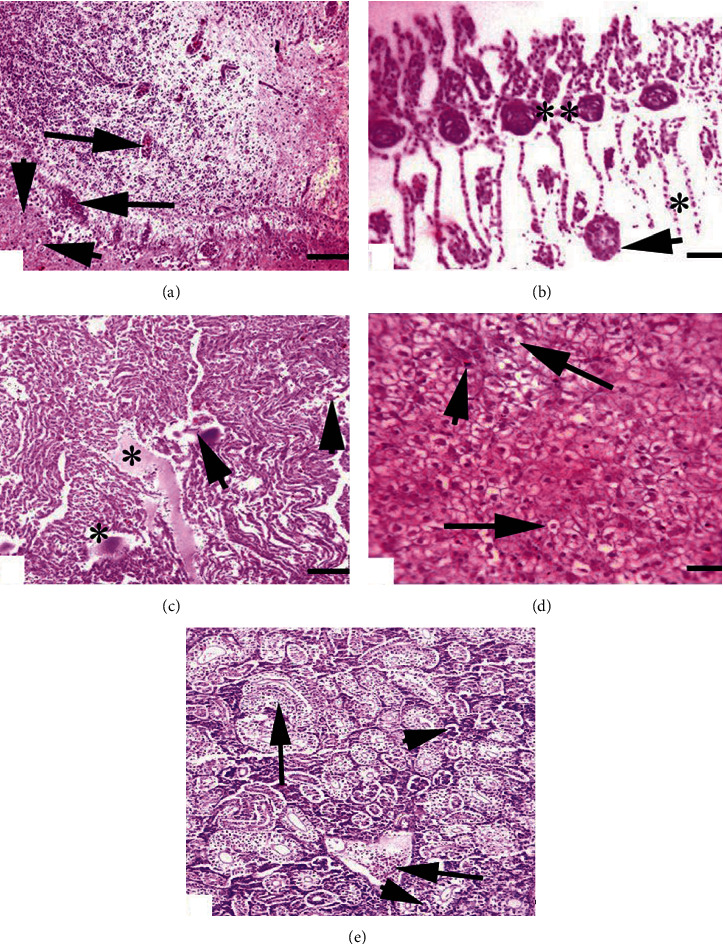
Photomicrograph of common carp (a) brain showing congestion (arrows) and necrosis of neurons (arrowhead). (b) Gills showing aneurysm (arrow), uplifting of lamellae (^∗^), and disruption of cartilaginous core (^∗∗^). (c) Heart showing edema (^∗^) and degeneration of cardiac muscles (arrowheads). (d) Liver showing atrophied hepatocyte (arrows) and necrotic hepatocyte (arrowhead). (e) Kidneys showing edema (arrows) and necrosis of tubules (arrowheads) to BPA at day 30 of study.

At necropsy level, no gross abnormalities were observed in visceral organs including the brain, gills, heart, kidneys, and liver of the untreated control group, and the fish kept in group B was exposed to the low concentration of BPA. Grossly, a dose- and time-dependent moderate to severe pathological lesions including degeneration and necrosis of neurons, edema and disorganization of cardiac muscles, loss of hepatocytes integrity, disruption of gill's primary and secondary lamellae, deformed renal tubules, and increased bowman space were observed in visceral organs (brain, gills, heart, kidneys, and liver) of fish kept in groups C and D. Histopathological observation of different sections of the brain of treated fish showed moderate to severe microscopic changes (Figures 1(a)–1(e)). Among few prominent changes including necrosis and degeneration of neurons, congestion and microgliosis were also observed in the brain of various treated fish (Figure 1(a)). Histopathological observations of the gills sections showed the moderate to severe uplifting and disruption of primary and secondary lamellae, fusion of secondary lamellae, congestion, disruption, and ruptured cartilaginous cord as well as severe hemorrhages shown in (Figure 1(b)). Disorganization of cardiac muscles, edema, and necrosis of cardiac cells was seen in heart's tissue sections (Figure 1(c)). Microscopic changes including increased bowman space, moderate to severe congestion, pyknosis, necrosis, and inflammation in tubular cells in kidney sections were evident in various treated groups of fish (Figure 1(e)). The degenerated hepatocytes, atrophy, cytoplasmic vacuolation, eccentric nuclei, fatty infiltration, necrosis, and congestion were observed in liver tissues of the treated fish (Figure 1(d)).

### 3.3. Morphological and Nuclear Changes in Erythrocytes

**Figure 2:**
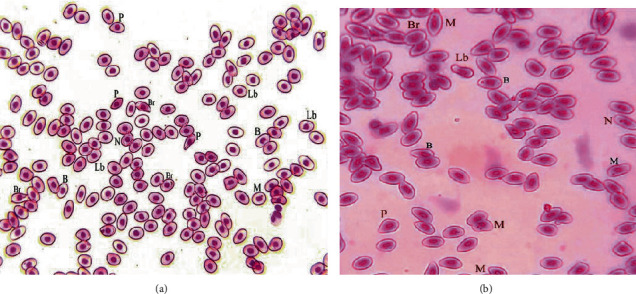
(a, b) Photograph of blood smear of common carp (*Cyprinus carpio*) fish exposed to BPA at day 30 showing different morphological and nuclear abnormalities in erythrocytes such as erythrocytes with lobed nucleus (Lb), erythrocytes with broken nucleus (BR), blabbed nucleus (B), nuclear remnants (N), Erythrocytes with micronucleus (M), and pear-shaped erythrocytes (P). Giemsa stain; ×1000.

Our study results clearly depicted the significant morphological changes in erythrocytes among treated groups (4.5 and 6 mg/L) (Table 2). The rate of different morphological and nuclear changes like pear shaped erythrocytes, leptocytes, microcytes, spherocytes, and erythrocytes with fragmented nucleus, lobed nucleus, micronucleus, blabbed nucleus, vacuolated nucleus, and nuclear remnants in red blood cells of fish exposed to 4.5 and 6 mg/L BPA exhibited significantly increased at day 30 as compared to the untreated group (Table 2). The values of erythrocytes with lobed, broken nucleus (BR), blabbed nucleus, nuclear remnants, micronucleus, and pear shaped erythrocytes significantly increased in fish exposed to 4.5 and 6 mg/L BPA at day 30 of the experiment (Figures 2(a) and 2(b)).

### 3.4. Oxidative Stress Parameters, Antioxidant Enzymes, and Genotoxicity

**Figure 3:**
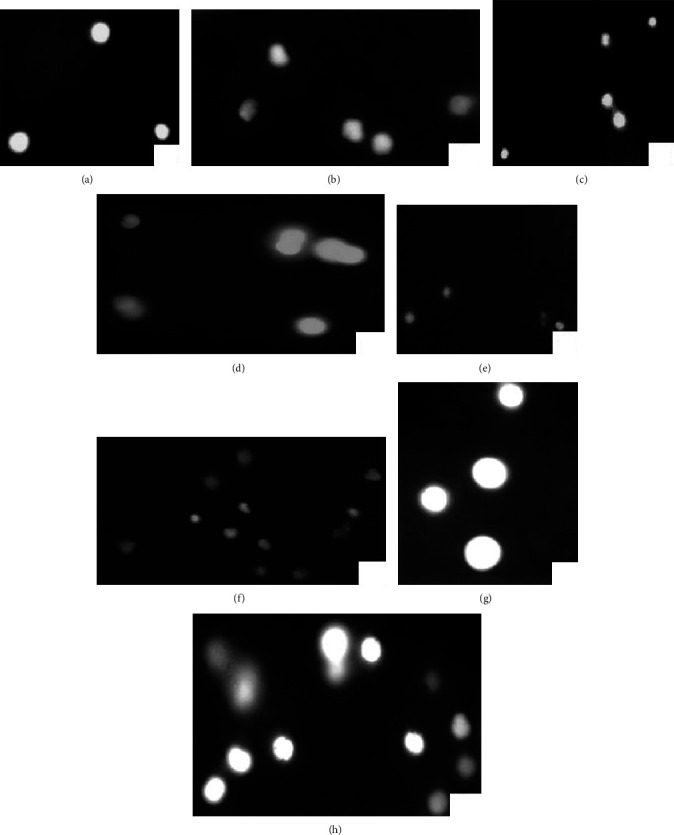
Photograph of comet assay/single cell electrophoresis showing (a) normal brain tissue cells with intact nuclear material, (b) brain tissue cells with damage nuclear material, (c) normal liver tissue cells with intact nuclear material, (d) liver tissue cells with damage nuclear material, (e) normal gills cells, (f) gill tissue cells with damage nuclear material, (g) normal kidney tissue cells, and (h) kidney tissue cells with damage nuclear material at day 30 displaying significant genotoxicity (DNA/nuclear damage) evident by fluorescing of nuclear material.

Current study results depicted the significantly increased generation of ROS and TBARS in brain tissue of fish treated with 4.5 and 6 mg/L concentrations of BPA at day 30 of trial (Table 3). The contents of TBARS and ROS increased significantly in brain tissue of treated fish. No significant differences were reported among the control group and fish exposed to 3 mg/L BPA. Antioxidants enzymes (SOD, CAT and POD) in our study were significantly reduced in brain tissue of fish treated to 4.5 and 6 mg/L concentrations of BPA (Table 3). Our study results displayed similar profile of significance regarding oxidative stress parameters and antioxidant enzymes in the liver, gills, and kidneys. All these organs showed significantly decreased values in fish groups treated to 4.5 and 6 mg/L concentrations of BPA at day 30 as compared to the fish untreated group. No significant results were reported regarding oxidative stress parameters and antioxidant enzymes to fish exposed to 3 mg/L BPA and control group (Table 3). Comet assay in current study (Figure 3) showed the DNA damage at day 30 in brain, liver, gills, and kidneys tissue of treated fish groups.

## 4. Discussion

Damaging extent of environmental toxins in aquatic life is of great importance. Various blood biochemical and histopathological changes may reflect the deleterious effects of pollutants on various exposed fish fauna [56, 57]. BPA, being an endocrine disruptor, has been applied to elucidate the toxic effects on different fish tissues like the brain, heart, gills, liver, and kidneys [12, 17, 58]. BPA continuous use and its emerging ill effects in the environment have fetch the attention of scientists to monitor the long-term effects at low dose exposures in order to minimize the risks to the public health. The innovative lay of the present study was the sublethality test of the BPA in *C. carpio* regarding clinical and histopathological changes in the vital organs. Primarily, some behavioral responses including movement of operculum, mucus secretion, irregular swimming pattern, trembling of fins, air gulping, body imbalance, and dark skin of fish were observed and compared with the control-A group. According to previous studies, these same observations were found comparable in *Cirrhinus mrigala* [59], *Labeo rohita* [12], *Ctenopharyngodon* [60], zebrafish [61], bighead carp [12], and *Channa punctatus* [62]. Moreover, clinical ailments in vertebrates [26, 63, 64] have also been reported. The findings of present study were observed which are also similar when *Heteropneustes fossilis* and *C. carpio* were exposed to different concentrations of insecticides [42, 65]. These behavioral changes in treated fish might be due to the learning deficits, neurotoxic effects, and irritation to the perceptive system of the animal's body [66–69].

According to hematological study, the values of hemoglobin, erythrocytes, lymphocytes, PCV, and monocytes were found significantly decreased depending on the dose concentration when compared with the control group, while gradually increased values of leucocytes and neutrophils were observed in treated groups when compared to the control group. Similar results were also reported when compared with the previous findings [57, 70]. They suggested that it might be due to very low supply of oxygen to RBCs. Hence, it has already been reported that different stress conditions in animals can increase the reactive oxygen species (ROS), white blood cells (WBCs), RBCs, Hb, and mean corpuscular hemoglobin concentration (MCHC), which is ultimately due to activation of the immune system, swelling and/or additional release of erythrocytes, decreased pH, and decreased plasma volume in the blood [71, 72]. The observed reduction in the hematological parameters may be due to the internal hemorrhage, destruction, and less production of erythrocytes due to toxic accumulation of BPA [73].

According to biochemical analysis of BPA exposed groups, the values of glucose, cholesterol, triglycerides, creatinine, and urea were observed significantly high as compared to the control group while albumin protein was observed gradually decreased in dose-dependent manner as compared to the control group. Various reports on other fish species are also available which indicate the same abnormal levels of urea and creatinine, damage of tissues of visceral organs, fatty liver, abnormal structure of cells, and malfunctioning of hepatic enzymes while exposed to BPA [66, 74]. Significant increase in creatinine and uric acid may indicate that BPA affects muscle and purine metabolism. This increase may also be due to the damage of renal tubules. The histopathological changes including degeneration and necrosis of glomerulus and decrease in hematopoietic tissue in the same fish species after BPA exposure were reported [17]. This decrease in hematopoietic tissue may be a cause of increase in serum uric acid. The lower values of these blood parameters could also be due to the hemolysis, rapid oxidation of hemoglobin, and destruction of erythrocyte [75, 76].

In the present study, the histopathological changes in gills like uplifting of primary and secondary lamellae, disruption of primary and secondary lamellae, fusion of secondary lamellae, congestion, and ruptured cartilaginous cord were observed in response of high dose of BPA. In the light of other studies, such major changes are responsible to increase the distance through which irritant can be reached to the blood stream; so, they could serve the defense mechanism against toxicant and may also support to enhance the ventilation capacity to compensate the impaired uptake of oxygen in fish [77]. Literature based on previous reports provide the same information on gills changes like sloughing of epithelium of primary and secondary lamella, lamellar stunting, curled lamellae arrangements, aneurysm, and edema in fish exposed to different toxic chemicals [42, 78, 79]. Different microscopic lesions in gills of zebrafish due to disruption of ionic regulation associated with BPA toxicity have also been reported [80].

In the present study, no histopathological changes were observed in fish liver of control groups. However, dose dependent severities including ruptured hepatocytes, ruptured central vein, necrosis, congestion, and degeneration in hepatocytes were observed in liver tissues of fish. Similar changes were observed when different levels of BPA were exposed to different fish species at different times [81, 82]. According to their findings, the fish exposed to various concentrations of BPA showed sinusoidal dilation, lipid accumulation, central vein congestion, necrosis, and hepatocyte vacuolization. Hence, same results have been reported with the exposure of another xenobiotic chemical nonylphenol to *Clarias gariepinus* [83]. In the light of previous studies, it is being suggested here that the observed changes in liver structure might be due to degeneration of structural proteins and accumulation of lipids in membranes. The vacuolization in hepatocytes is also due to the improper synthesis of substances in parenchymal cells and its release into blood circulation [84].

No histological changes were observed in the kidney section in the control group; hence, dose-dependent effects like necrosis and inflammation in tubular cells, degeneration in renal cells, congestion, and increased bowman space were observed. More or less, these findings can be compared in *C. catla* [17] and *Heteropneustes fossilis* [33] when exposed to BPA. They reported hypertrophy of glomerulus, degeneration, and dissociation of renal tubules and bowman capsule, proliferation in the renal tubule and haemopoietic tissue, shrinkage of glomerulus, pyknosis, dilated blood vessel, rupture of bowman capsule, and obliterated bowman space on sublethal exposure BPA. Same results were also observed with the exposure of other chemicals [85].

In this present study, the histopathological observation of different sections of the brain of treated fish showed few prominent microscopic changes including necrosis and degeneration of neurons, congestion, and microgliosis in brain tissues of various treated fish. Basically, these microscopic changes were due to gradual increase in lipid peroxidation and increased stress biomarkers (ROSs). However, it is well documented in previous reports that exposure of animals to different toxicants causes detoxification in their bodies by increasing the level of ROS which ultimately results in less production of antioxidant enzymes [12, 26, 86]. Previously toxic effects of BPA on brain of fish have also been reported [87]. According to previous studies, BPA may cause neuroendocrine disruption by altering the mechanism of kisspeptin signaling pathways [88] and by also down regulating the genes involved in dopaminergic processes [24].

In this study, the histopathological sections of fish heart indicated the disorganization of cardiac muscles, edema, and necrosis of cardiac cells in the BPA exposed fish. Literature on potential toxic effects of BPA on fish heart is limited; however, a few studies were showing likewise results as hemorrhages, edema, neutrophilic myocarditis, and accumulation of fibrin in bighead carp [12]. According to another report on zebrafish, a high level exposure of BPA caused abnormalities in structure and function of heart like abnormal curvature caused low ventricular beat rate and blood flow and also caused calcific aortic valve disease with extra cellular matric in the heart [89]. It has also been reported that cardiovascular tissues have estrogen receptors which make heart more susceptible to endocrine disrupter BPA which may bring severe pathological changes in different tissues by altering the estrogenic pathways in the body [90].

Our study results showed significant altered nuclear morphology in erythrocytes of *C. carpio* including different abnormalities like fragmented, lobed, blabbed, vacuolated, and micronucleus. Morphological abnormalities of erythrocytes like pear shaped, leptocytes, microcytes, and spherocytes were also reported in current study exposed to BPA toxicity. Similar results were also documented earlier in bighead carp due to BPA toxicity [12]. These morphological abnormalities in erythrocytes and nuclear changes can be best ascribed to oxidative stresses in erythrocytes of fish [12, 26]. Morphological and nuclear alterations in erythrocytes of fish can be attributed to BPA interaction with receptors, lipid peroxidation, and debilitated function of mitochondria [91]. Current study illustrated significantly heightened oxidative stress parameters and lower antioxidant enzymes in the brain, liver, gills, and kidneys of BPA treated fish. Similar results were reported previously in bighead carp fish [12]. Different organism exposed to environmental toxins show increased generation of ROS occurs due to detoxifying mechanisms. Production of ROS generally based upon BPA concentration and its duration lead to lipid peroxidation process which ultimately results in cell membrane irregularities and increased production of TBARS [82, 92]. Higher values of oxidative stress contents in present study might be due to exhaustion and imbalance of antioxidants enzymes that are best supported by documented reports [12, 93]. Oxidative stress induced by BPA in target organisms lead to reduced generation of antioxidants enzymes and enhanced lipid peroxidation [14, 94].

Our results disclosed reduced antioxidant contents (SOD, CAT, POD) in the brain, liver, gills, and kidneys of treated fish. Our results are in accordance to reported studies [12, 95]. Decrease in antioxidants contents in the treated fish group can be described due to malfunctional tissues and enhanced consumption of energy to cope the oxidative stresses. Not too much information is available regarding the antioxidants parameters in common carp exposed to BPA. The decreased concentration of antioxidants enzymes in different tissues in current study can be best ascribed due to heightened oxidative stresses and reduction of antioxidants in these tissues which are supported by reported studies [82, 86, 96]. The decreased concentration of antioxidants in the brain, liver, gills and kidneys in the current study could be due to increased generation of free radicals in these tissues due to BPA that leads to atypical functions and disturbance of antioxidants processes [53, 82].

In the current study, comet assay results displayed significant genotoxicity (nuclear damage) in isolated liver, brains, gills, and kidney tissues. It is well known that comet assay is the approach that is widely accepted and used to evaluate the nuclear damages in different tissues of aquatic organisms [97–99]. In literature, no significant reports are available regarding the nuclear damage in common carp so far but few reports have been documented about nuclear damage to BPA in zebrafish [100] and bighead carp [12]. Genotoxicity (nuclear damage) in common carp in our study may be attributed to elevated generation of free radicals and oxidative stresses. However, the detailed underlying mechanism at cell and molecular level is still not clear. However, genotoxicity induced in response to BPA exposure can be described as oxidative stresses through ROS and lipid peroxidation [101] which can lead to nuclear anomalies [88]. However, current study speculations related to nuclear damage in different tissues of common carp may be attributed to genetic alterations in exposure to BPA triggering to abnormal functioning of proteins accountable for mitochondria malfunctioning and nuclear proteins fragmentation. Various previous studies have indicated that BPA causes genotoxic effects due to induction of oxidative stress through rapid generation of free radicals and lipid peroxidation [12, 102, 103].

## 5. Conclusion

Conclusively, the main mechanism involved in hematobiochemical and histopathological modifications in fish was due to the gradual increase of oxidative stress caused by BPA. The findings obtained from this research are primarily valuable to monitor the sublethal effects of the chemical on a prolific breeder *C. carpio*. Our study depicted that BPA causes adverse effects on erythrocytes and different tissues of common carp. This study also clearly displayed genotoxicity (nuclear damage) in all isolated tissues of common carp subjected to BPA exposure. Furthermore, induced BPA toxicity causes heightened oxidative stresses and reduced antioxidants enzymes activities in the brain, liver, gills, and kidneys of common carp leading to dysfunction and altered tissue histology. Current study will highlight a key concern about the human health directly or indirectly due to bisphenol toxicity exposure of aquatic animals especially common carp, which are of great economic value and of dietary importance.

## Figures and Tables

**Table 1:** Hematobiochemical profile of fish exposed to different concentrations of BPA.

Parameters	Treatment groups			
	Control A	B (3 mg/L)	C (4.5 mg/L)	D (6 mg/L)
Red blood cells (10^6^/mm^3^)	4.42 ± 0.10	3.92 ± 0.09	3.22 ± 0.06^∗^	2.15 ± 0.09^∗^
Hemoglobin (g/dL)	9.95 ± 0.78	9.40 ± 0.10	7.95 ± 0.01^∗^	6.45 ± 0.09^∗^
Pack cell volume (%)	40.92 ± 2.16	38.89 ± 1.15	38.10 ± 0.13^∗^	29.51 ± 0.55^∗^
White blood cells (10^3^/mm^3^)	15.25 ± 0.34	18.68 ± 0.05	19.05 ± 0.18^∗^	25.92 ± 0.94^∗^
Neutrophil (%)	15.35 ± 0.18	18.90 ± 0.71	19.29 ± 0.89^∗^	23.92 ± 0.91^∗^
Lymphocyte (%)	21.23 ± 0.12	19.91 ± 0.09	18.83 ± 0.06^∗^	15.50 ± 0.70
Monocyte (%)	3.38 ± 0.09	3.09 ± 0.08	3.05 ± 0.06^∗^	2.14 ± 0.01^∗^
Glucose (mg/dL)	37.87 ± 1.66	38.87 ± 1.66	42.12 ± 1.27^∗^	44.98 ± 1.51^∗^
Cholesterol (mg/dL)	183.53 ± 1.38	187.61 ± 1.33	190.51 ± 1.21^∗^	212.75 ± 1.27^∗^
Triglycerides (mg/dL)	192.01 ± 1.71	193.11 ± 1.81	196.22 ± 1.91^∗^	220.08 ± 1.81^∗^
Albumin (mg/dL)	3.42 ± 0.11	3.40 ± 0.11	3.15 ± 0.08^∗^	2.45 ± 0.05^∗^
Creatinine (mg/dL)	1.82 ± 0.02	1.87 ± 0.01	2.11 ± 0.02^∗^	2.38 ± 0.01^∗^
Urea (mg/dL)	11.31 ± 0.33	12.38 ± 0.34	12.95 ± 0.01^∗^	16.99 ± 0.01^∗^

The data are represented as mean ± SD. Values bearing asterisk in each rows show significant difference as compared to the control group (*P* < 0.05).

**Table 2:** Morphological and nuclear changes in erythrocytes of fish exposed to different concentrations of BPA.

Parameters	Treatment groups			
	Control A	B (3 mg/L)	C (4.5 mg/L)	D (6 mg/L)
Morphological changes in erythrocytes				
Pear shaped erythrocyte (%)	0.59 ± 0.03	0.61 ± 0.03	0.63 ± 0.03^∗^	0.93 ± 0.02^∗^
Leptocytes (%)	0.38 ± 0.02	0.39 ± 0.02	0.84 ± 0.2^∗^	0.98 ± 0.02^∗^
Microcytes (%)	0.60 ± 0.01	0.64 ± 0.01	0.88 ± 0.01^∗^	0.97 ± 0.01^∗^
Spherocytes (%)	0.40 ± 0.03	0.42 ± 0.03	1.64 ± 0.03^∗^	2.76 ± 0.03^∗^
Erythrocyte with broken nucleus (%)	0.28 ± 0.01	0.32 ± 0.01	0.96 ± 0.01^∗^	1.40 ± 0.01^∗^
Erythrocyte with lobed nucleus (%)	0.41 ± 0.01	0.45 ± 0.01	1.49 ± 0.01^∗^	1.53 ± 0.01^∗^
Erythrocyte with micronucleus (%)	0.38 ± 0.03	0.39 ± 0.03	2.62 ± 0.03^∗^	3.74 ± 0.03^∗^
Erythrocyte with blabbed nucleus (%)	0.26 ± 0.02	0.28 ± 0.02	0.94 ± 0.02^∗^	1.50 ± 0.02^∗^
Erythrocyte with vacuolated nucleus (%)	0.17 ± 0.01	0.21 ± 0.01	0.22 ± 0.01^∗^	1.29 ± 0.01^∗^
Erythrocyte with nuclear remnants (%)	0.22 ± 0.01	0.24 ± 0.01	0.27 ± 0.01^∗^	1.34 ± 0.01^∗^

The data are represented as mean ± SD. Values bearing asterisk in each rows show significant difference as compared to the control group (*P* < 0.05).

**Table 3:** Oxidative stress parameters (ROS, TBARS, GSH) and quantity of antioxidant enzymes (SOD, CAT, POD) in the brain, liver, gills, and kidneys of fish exposed to different concentrations of BPA.

Parameters	Treatment groups			
	Control A	B (3 mg/L)	C (4.5 mg/L)	D (6 mg/L)
Brain				
Reactive oxygen species (ROS)contents (optical density)	0.23 ± 0.03	0.25 ± 0.05	0.31 ± 0.01^∗^	0.35 ± 0.02^∗^
Thiobarbituric acid reactive substances (TBARS) content (nmol/TBARS formed/mg protein/min)	0.26 ± 0.03	0.28 ± 0.04	0.36 ± 0.01^∗^	0.37 ± 0.03^∗^
Reduced glutathione GSH (*μ*mol/g tissue)	2.31 ± 0.01	2.25 ± 0.01	1.79 ± 0.11^∗^	1.73 ± 0.09^∗^
Antioxidant enzymes				
Superoxide dismutase SOD (units/mg protein)	9.33 ± 0.22	9.31 ± 0.26	7.39 ± 0.25^∗^	7.27 ± 0.23^∗^
Catalase CAT (units/min)	4.32 ± 0.15	4.24 ± 0.18	3.28 ± 0.27^∗^	3.16 ± 0.02^∗^
Peroxidase POD (units/min)	2.76 ± 0.09	2.68 ± 0.06	2.04 ± 0.01^∗^	2.01 ± 0.03^∗^
Liver				
Reactive oxygen species (ROS)contents (optical density)	0.17 ± 0.01	0.21 ± 0.02	0.25 ± 0.01^∗^	0.29 ± 0.05^∗^
Thiobarbituric acid reactive substances (TBARS) content (nmol/TBARS formed/mg protein/min)	25.6 ± 0.19	25.9 ± 0.29	29.2 ± 0.09^∗^	32.5 ± 0.17^∗^
Reduced glutathione GSH (*μ*mol/g tissue)	5.63 ± 0.01	5.57 ± 0.06	5.51 ± 0.01^∗^	4.55 ± 0.01^∗^
Antioxidant enzymes				
Superoxide dismutase SOD (units/mg protein)	12.17 ± 0.19	12.07 ± 0.13	10.37 ± 0.19^∗^	9.07 ± 0.21^∗^
Catalase CAT (units/min)	6.49 ± 0.12	6.45 ± 0.07	4.26 ± 0.02^∗^	4.16 ± 0.05^∗^
Peroxidase POD (units/min)	4.73 ± 0.07	4.51 ± 0.09	3.49 ± 0.02^∗^	3.37 ± 0.01^∗^
Gills				
Reactive oxygen species (ROS)contents (optical density)	0.19 ± 0.03	0.23 ± 0.02	0.27 ± 0.01^∗^	0.31 ± 0.01^∗^
Thiobarbituric acid reactive substances (TBARS) content (nmol/TBARS formed/mg protein/min)	34.62 ± 0.21	35.30 ± 0.21	39.98 ± 0.11^∗^	40.66 ± 0.23^∗^
Reduced glutathione GSH (*μ*mol/g tissue)	1.33 ± 0.02	1.27 ± 0.03	1.07 ± 0.03^∗^	1.02 ± 0.03^∗^
Antioxidant enzymes				
Superoxide dismutase SOD (units/mg protein)	8.87 ± 0.05	8.65 ± 0.07	7.03 ± 0.02^∗^	7.01 ± 0.03^∗^
Catalase CAT (units/min)	2.90 ± 0.01	2.78 ± 0.02	2.06 ± 0.05^∗^	2.01 ± 0.03^∗^
Peroxidase POD (units/min)	0.32 ± 0.03	0.28 ± 0.01	0.230 ± 0.05^∗^	0.19 ± 0.04^∗^
Kidneys				
Reactive oxygen species (ROS)contents (optical density)	0.32 ± 0.06	0.36 ± 0.01	0.40 ± 0.03^∗^	0.44 ± 0.07^∗^
Thiobarbituric acid reactive substances (TBARS) content (nmol/TBARS formed/mg protein/min)	34.15 ± 0.23	36.87 ± 0.23	39.59 ± 0.21^∗^	40.31 ± 0.23^∗^
Reduced glutathione GSH (*μ*mol/g tissue)	4.42 ± 0.01	4.22 ± 0.04	3.35 ± 0.11^∗^	3.25 ± 0.09^∗^
Antioxidant enzymes				
Superoxide dismutase SOD (units/mg protein)	12.50 ± 0.11	11.91 ± 0.13	9.22 ± 0.18^∗^	9.507 ± 0.13^∗^
Catalase CAT (units/min)	4.23 ± 0.07	4.17 ± 0.05	3.31 ± 0.11^∗^	3.15 ± 0.03^∗^
Peroxidase POD (units/min)	3.96 ± 0.02	3.84 ± 0.02	3.12 ± 0.09^∗^	3.02 ± 0.07^∗^

The data are represented as mean ± SD. Values bearing asterisk in each rows show significant difference as compared to control group (*P* < 0.05).

## Data Availability

No data were used to support this study.
